# *Dehalogenimonas* spp. can Reductively Dehalogenate High Concentrations of 1,2-Dichloroethane, 1,2-Dichloropropane, and 1,1,2-Trichloroethane

**DOI:** 10.1186/2191-0855-2-54

**Published:** 2012-10-09

**Authors:** Andrew D Maness, Kimberly S Bowman, Jun Yan, Fred A Rainey, William M Moe

**Affiliations:** 1 Department of Civil and Environmental Engineering, Louisiana State University, 3513B Patrick Taylor Hall, Baton Rouge, LA, 70803, USA; 2 Department of Biological Sciences, Louisiana State University, Baton Rouge, LA, 70803, USA; 3 Department of Biological Sciences, University of Alaska Anchorage, Anchorage, AK, 99508, USA; 4 Present address: Jun Yan, Department of Microbiology and Department of Civil and Environmental Engineering, University of Tennessee, Knoxville, TN, USA

**Keywords:** Bioremediation, Chlorinated alkanes, Dehalogenimonas, Reductive dechlorination, Dehalogenation

## Abstract

The contaminant concentrations over which type strains of the species *Dehalogenimonas alkenigignens* and *Dehalogenimonas lykanthroporepellens* were able to reductively dechlorinate 1,2-dichloroethane (1,2-DCA), 1,2-dichloropropane (1,2-DCP), and 1,1,2-trichloroethane (1,1,2-TCA) were evaluated. Although initially isolated from an environment with much lower halogenated solvent concentrations*, D. alkenigignens* IP3-3^T^ was found to reductively dehalogenate chlorinated alkanes at concentrations comparable to *D. lykanthroporepellens* BL-DC-9^T^. Both species dechlorinated 1,2-DCA, 1,2-DCP, and 1,1,2-TCA present at initial concentrations at least as high as 8.7, 4.0, and 3.5 mM, respectively. The ability of *Dehalogenimonas* spp. to carry out anaerobic reductive dechlorination even in the presence of high concentrations of chlorinated aliphatic alkanes has important implications for remediation of contaminated soil and groundwater.

## Introduction

In industry, polychlorinated ethanes and propanes are used as solvents, degreasing agents, and paint removers; they are also globally produced on a massive scale as intermediates during production of other industrially important chemicals ([De Wildeman and Verstraete, 2003]; [Field and Sierra-Alvarez, 2004]). Due to spills and past disposal methods, these chlorinated organic compounds are prevalent groundwater and soil contaminants. For example, 1,2-dichloroethane (1,2-DCA) is present in at least 570 current or former Superfund sites [(ATSDR 2001]), and 1,2-dichloropropane (1,2-DCP) is present at more than 100 Superfund sites ([Fletcher et al., 2009]). The prevalence of these polychlorinated alkanes as environmental contaminants is of concern because of their known or suspected toxicity and/or carcinogenicity ([ATSDR, 2001]; [1989]).

Anaerobic reductive dechlorination, a process in which microorganisms utilize chlorinated organics as electron acceptors, represents a potentially viable method for cleanup of many contaminated sites ([Christ et al., 2005]; [Fennell et al., 2001]; [He et al., 2005]; [Major et al., 2002]). Previous studies on reductive dechlorination of halogenated alkanes have generally been conducted in a relatively narrow range of low (e.g., 0.1 to 0.5 mM) contaminant concentrations ([Chen et al., 1996]; [De Wildeman et al., 2003]; [Fletcher et al., 2009]; [Grostern and Edwards, 2006,^2009^]; [Lorah and Olsen, 1999]; [Maymó-Gatell et al., 1999]; [Yan et al., 2009a]). Contaminant concentrations considerably higher than this range are present at some sites, however, particularly in areas where pollutants remain in the subsurface as dense non-aqueous-phase liquids (DNAPLs) ([Bowman et al., 2006]; [Marzorati et al., 2007]; [Yan et al., 2009b]).

Among the limited number of microbes known to anaerobically reductively dehalogenate polychlorinated ethanes and propanes are strains of *Dehalogenimonas lykanthroporepellens* ([Moe et al., 2009]; [Yan et al., 2009a]) and *Dehalogenimonas alkenigignens* ([Bowman et al., 2012]). These species cluster in the phylum *Chloroflexi*, related to but distinct from organohalide respiring *Dehalococcoides* strains ([Bowman et al., 2012]; [Löffler et al., 2012]; [Moe et al., 2009]). Strains of both *Dehalogenimonas* species reductively dehalogenate 1,2-DCA, 1,2-DCP, and 1,1,2-TCA via dichloroelimination reactions with H_2_ as an electron donor, forming final products of ethene, propene, and vinyl chloride, respectively ([Bowman et al., 2012]; [Yan et al., 2009a]).

Previously reported studies of *Dehalogenimonas* strains were conducted only at initial chlorinated solvent concentrations of 0.5 mM ([Bowman et al., 2012]; [Yan et al., 2009a]). Research reported here was aimed at evaluating the solvent concentration ranges over which *D. lykanthroporepellens* and *D. alkenigignens* can reductively dechlorinate 1,2-DCA, 1,2-DCP, and 1,1,2-TCA in order to assess their suitability for bioremediation of high contaminant concentrations.

## Materials and methods

Experiments were carried out in 25 mL glass serum bottles (Wheaton) sealed with butyl rubber stoppers and aluminum crimp caps. Each serum bottle contained 10 mL titanium-citrate reduced anaerobic basal medium prepared as described by ([Moe et al. 2009]) except that 5 mM acetate was replaced with 0.05 mM each of acetate, pyruvate, and lactate. The 15 mL gas headspace was comprised of H_2_/N_2_ (80%/20%, v/v). Replicate serum bottles were spiked with neat, filter sterilized 1,2-DCA (>99.8% purity, Sigma Aldrich, St. Louis, MO), 1,2-DCP (99%, Sigma Aldrich, St. Louis, MO), or 1,1,2-TCA (96%, Sigma Aldrich, St. Louis, MO) to achieve target initial aqueous phase concentrations ranging from 0.5 to 15 mM after dissolution and equilibration.

Each serum bottle received 0.3 mL inoculum (3% v/v) of *D. alkenigignens* strain IP3-3^T^ (=JCM 17062^T^ =NRRL B-59545^T^) or *D. lykanthroporepellens* strain BL-DC-9^T^ (=JCM 15061^T^ = ATCC BAA-1523^T^) previously grown on 1,2-DCP. Incubation was in the dark at 30^o^C without shaking. Triplicate bottles were sacrificed at time zero and after eight weeks incubation for analysis of chlorinated solvents and potential degradation products. To account for potential abiotic reactions, triplicate negative controls prepared in the same manner as inoculated bottles but without bacterial addition were incubated under identical conditions.

Chlorinated solvents and degradation products were measured using an HP model 6890 gas chromatograph (GC) equipped with a flame ionization detector and GS-GasPro capillary column (60 m × 0.32 mm I.D., J&W P/N 113–4362) as described previously ([Yan et al., 2009a]). Gas headspace samples collected in 100 μL gas-tight glass syringes (Hamilton, Baton Rouge, LA) were introduced to the GC via direct injection. Aqueous samples (500 μL) were introduced to the GC via a Tekmar 2016/3000 purge and trap autosampler and concentrator. Both gas-headspace and aqueous-phase aliquots were analyzed for each sample bottle.

Hydrogen concentrations in the gas headspace were measured using an SRI Instruments model 310 gas chromatograph (Torrence, CA) equipped with a thermal conductivity detector and molecular sieve column (Alltech Molesieve 5A 80/100) as described previously ([van Ginkel et al., 2001]).

## Results

**Figure 1:**
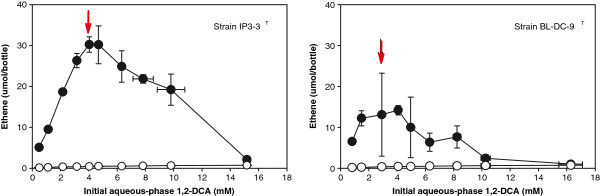
**Experimentally measured ethene production as a function of initial aqueous-phase 1,2-DCA concentration after eight-weeks incubation of*D. alkenigignens*IP3-3**^**T**^**(left) and*D. lykanthroporepellens*BL-DC-9**^**T**^**(right).** Filled symbols indicate average of replicate bottles inoculated with bacterial strains. Open symbols indicate average of replicate uninoculated negative control bottles. Bars represent one standard deviation. Arrows denote concentration at and above which >1% of the starting 1,2-DCA remained at the end of the incubation period.

**Figure 2:**
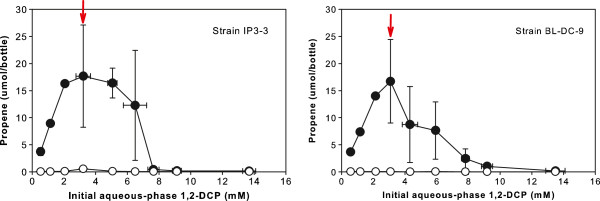
**Experimentally measured propene production as a function of initial aqueous-phase 1,2-DCP concentration after eight-weeks incubation of*D. alkenigignens*IP3-3**^**T**^**(left) and*D. lykanthroporepellens*BL-DC-9**^**T**^**(right).** Filled symbols indicate average of replicate bottles inoculated with bacterial strains. Open symbols indicate average of replicate uninoculated negative control bottles. Bars represent one standard deviation. Arrows denote concentration at and above which >1% of the starting 1,2-DCP remained at the end of the incubation period.

**Figure 3:**
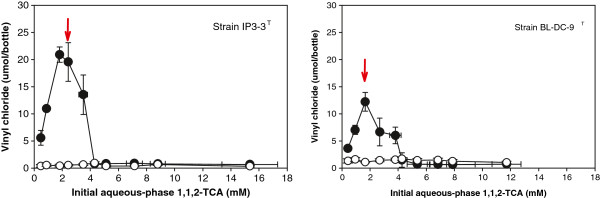
**Experimentally measured vinyl chloride production as a function of initial aqueous-phase 1,1,2-TCA concentration after eight-weeks incubation of*D. alkenigignens*IP3-3**^**T**^**(left) and*D. lykanthroporepellens*BL-DC-9**^**T**^**(right).** Filled symbols indicate average of replicate bottles inoculated with bacterial strains. Open symbols indicate average of replicate uninoculated negative control bottles. Bars represent one standard deviation. Arrows denote concentration at and above which >1% of the starting 1,1,2-TCA remained at the end of the incubation period.

The quantity of the dechlorination product determined at the end of the eight week incubation period as a function of initial aqueous-phase 1,2-DCA, 1,-DCP, and 1,1,2-TCA is shown in Figures 1, 2 and 3 respectively.

The production of ethene (Figure 1) coupled with 1,2-DCA disappearance in the inoculated bottles is consistent with the 1,2-DCA dihaloelimination degradation pathway reported previously for *D. alkenigignens* IP3-3^T^ and *D. lykantroporepellens* BL-DC-9^T^ in tests conducted with initial 1,2-DCA concentrations of 0.5 mM in serum bottles containing H_2_ at an initial concentration of 10% v/v (as opposed to the 80% v/v employed in the present study) ([Bowman et al., 2012]; [Yan et al., 2009a]). Trace levels of 1-chloroethane (<0.3 μmol/bottle) were detected at comparable levels in both inoculated bottles and in uninoculated abiotic controls (data not shown) and small amounts of ethene (<0.7 μmol/bottle) were detected in abiotic negative controls (Figure 1) indicating that some abiotic 1,2-DCA transformation occurred in the anaerobic medium employed here, but the amount was negligible. The sum of parent compound (i.e., 1,2-DCA) plus daughter product (i.e., ethene and 1-chloroethane) in replicate serum bottles inoculated with the bacterial strains ranged from 74-107% of the mass determined in abiotic negative controls (average 89%). Dechlorination was essentially complete (< 1% of the starting 1,2-DCA remaining) at the end of the eight week incubation period for serum bottles supplemented with 1,2-DCA at initial concentrations less than 3.16 ± 0.05 mM and 1.48 ± 0.03 mM (mean ± standard deviation) for *D. alkenigignens* IP3-3^T^ and *D. lykanthroporepellens* BL-DC-9^T^, respectively (Figure 1). At higher initial 1,2-DCA concentrations (at and to the right of concentrations denoted by arrows in Figure 1), untransformed 1,2-DCA remained at the end of the eight week incubation in amounts increasing with increasing initial 1,2-DCA concentration.

The quantity of ethene observed increased with increasing initial 1,2-DCA concentration in the range of 0.5 to approximately 4 mM (maximum ethene observed in bottles containing initial 1,2-DCA concentrations of 4.03 ± 0.09 and 4.08 ± 0.16 mM for *D. alkenigignens* IP3-3^T^ and *D. lykanthroporepellens* BL-DC-9^T^, respectively) and then decreased at higher initial 1,2-DCA concentrations. The decrease in ethene production as 1,2-DCA concentrations increased indicates that sufficiently high 1,2-DCA concentrations can inhibit dechlorination activity of both *Dehalogenimonas* spp. Biologically mediated 1,2-DCA reductive dechlorination, however, was observed in serum bottles with initial 1,2-DCA concentrations as high as 9.81 ± 0.98 and 8.69 ± 0.26 mM for *D. alkenigignens* IP3-3^T^ and *D. lykanthroporepellens* BL-DC-9^T^, respectively. At higher initial 1,2-DCA concentrations, small amounts of ethene were also detected, but in amounts that were not statistically different from abiotic negative controls at a confidence level of 95%.

The production of propene (Figure 2) coupled with 1,2-DCP dechlorination in the inoculated bottles is consistent with the previously reported tests conducted with 0.5 mM 1,2-DCP in serum bottles with 10% v/v H_2_ in the gas headspace ([Bowman et al., 2012]; [Yan et al., 2009a]). Trace levels of 1-chloropropane (<0.03 μmol/bottle) were detected in inoculated bottles and uninoculated abiotic controls (data not shown), and propene was detected in relatively minute quantities (<0.13 μmol/bottle) in abiotic negative controls (Figure 2), indicating small amounts of abiotic 1,2-DCP transformation. The sum of parent chlorinated solvent (i.e., 1,2-DCP) and daughter products (i.e., propene and 1-chloropropane) in replicate bottles inoculated with the bacterial strains ranged from 74-131% of the mass determined in abiotic negative controls (average 95%). When provided with 1,2-DCP at initial aqueous-phase concentrations less than 3.19 ± 0.20 mM and 2.14 ± 0.12 mM, dechlorination of 1,2-DCP to a final product of propene was essentially complete in bottles inoculated with *D. alkenigignens* IP3-3^T^ and *D. lykanthroporepellens* BL-DC-9^T^, respectively, with <1% of the starting 1,2-DCP remaining at the end of the eight week incubation period (Figure 2). At higher initial 1,2-DCP concentrations (denoted by arrows in Figure 2), 1,2-DCP remained at the end of eight weeks in amounts increasing with increasing initial 1,2-DCP concentration.

Similar to what was observed with 1,2-DCA, the quantity of propene formed from 1,2-DCP dechlorination increased at initial 1,2-DCP concentrations ranging from 0.5 to roughly 3 mM (maximum propene was observed in bottles containing initial 1,2-DCP concentrations of 3.21 ± 0.46 and 3.08 ± 0.05 mM for *D. alkenigignens* IP3-3^T^ and *D. lykanthroporepellens* BL-DC-9^T^, respectively) and then decreased at higher initial 1,2-DCP concentrations. This indicates that beyond a certain threshold, as was observed with 1,2-DCA, 1,2-DCP became inhibitory to dechlorination activity. Nevertheless, 1,2-DCP reductive dechlorination was observed in serum bottles with initial 1,2-DCP concentrations as high as 5.05 ± 0.29 and 4.02 ± 0.09 mM for *D. alkenigignens* IP3-3^T^ and *D. lykanthroporepellens* BL-DC-9^T^, respectively. At higher initial 1,2-DCP concentrations, propene was also detected, but in amounts that were not statistically different from abiotic negative controls at a 95% confidence level.

In contrast to the relatively high concentrations of 1,2-DCP that were dechlorinated by *Dehalogenimonas* spp. in the present study, ([Löffler et al. 1997]) reported that 1,2-DCP dechlorination by an undefined mixed culture derived from Red Cedar Creek sediment (Michigan, USA) was completely inhibited when 1,2-DCP was supplied in amounts corresponding to an aqueous phase concentration of roughly 0.9 mM or higher. *D. alkenigignens* IP3-3^T^ and *D. lykanthroporepellens* BL-DC-9^T^ may be better suited to degradation of higher 1,2-DCP concentrations than other microbial populations studied previously.

Vinyl chloride production (Figure 3) coupled with 1,1,2-TCA dechlorination in the inoculated bottles is consistent with the previously reported tests conducted with 0.5 mM 1,1,2-TCA in serum bottles with 10% v/v H_2_ in the gas headspace ([Bowman et al., 2012]; [Yan et al., 2009a]). Low levels of 1,2-DCA (< 1.5 μmol/bottle) were observed in both inoculated bottles and uninoculated negative controls (data not shown), and small quantities vinyl chloride (<1 μmol/bottle) were observed in abiotic negative controls (Figure 3), indicating some abiotic 1,1,2-TCA transformation but in comparatively small amounts. The sum of the parent solvent (i.e., 1,1,2-TCA) and the daughter products (i.e., 1,2-DCA and vinyl chloride) in replicate bottles inoculated with the bacterial strains ranged from 74-146% of the mass determined in abiotic negative controls (average 99%). Dechlorination was essentially complete (<1% 1,1,2-TCA remaining) after 8 weeks incubation when *D. alkenigignens* IP3-3^T^ and *D. lykanthroporepellens* BL-DC-9^T^ were supplied with initial 1,1,2-TCA aqueous-phase concentrations below 2.42 ± 0.22 mM and 1.65 ± 0.03 mM, respectively (Figure 3). At higher initial concentrations, untransformed 1,1,2-TCA remained at the end of the incubation period.

Similar to what was observed with 1,2-DCA and 1,2-DCP, the quantity of vinyl chloride formed from 1,1,2-TCA dechlorination increased at initial 1,1,2-TCA concentrations ranging from 0.5 to roughly 2 mM and then decreased at higher initial 1,1,2-TCA concentrations (Figure 3). Maximum vinyl chloride concentrations were observed in bottles containing initial 1,1,2-TCA concentrations of 1.82 ± 0.18 and 1.65 ± 0.03 mM for *D. alkenigignens* IP3-3^T^ and *D. lykanthroporepellens* BL-DC-9^T^, respectively. This indicates that beyond a certain threshold, as was observed with 1,2-DCA and 1,2-DCP, 1,1,2-TCA became inhibitory to dechlorination activity. Nevertheless, biologically mediated 1,1,2-TCA reductive dechlorination was observed in serum bottles with initial 1,1,2-TCA concentrations as high as 3.49 ± 0.31 and 3.80 ± 0.42 mM for *D. alkenigignens* IP3-3^T^ and *D. lykanthroporepellens* BL-DC-9^T^, respectively. At higher initial 1,1,2-TCA concentrations, small amounts of vinyl chloride were also detected, but in amounts that were not statistically different from abiotic negative controls at a confidence level of 95%.

Hydrogen (H_2_) remained at relatively high concentrations (>62%, v/v) in the gas headspace at the end of the eight-week incubation period for all chlorinated solvent concentrations tested for both strains, indicating that it was not stoichiometrically limiting.

## Discussion

As a basis for comparing the concentrations tested here relative to saturation concentrations, solubility in water at 20°C is 86.1 mM for 1,2-DCA ([Horvath et al., 1999]), 23.9 mM for 1,2-DCP ([Horvath et al., 1999]), and 32.9 mM for 1,1,2-TCA (ATSDR [1989]). Also as a basis for comparison, groundwater in the well from which *D. lykanthroporepellens* BL-DC-9^T^ was isolated had 1,2-DCA, 1,2-DCP, and 1,1,2-TCA concentrations that averaged 5.5 mM, 0.6 mM, and 2.8 mM, respectively ([Bowman et al., 2006]; [Yan et al., 2009a]). Results determined here indicate that both *D. alkenigignens* IP3-3^T^ and *D. lykanthroporepellens* BL-DC-9^T^ can reductively dehalogenate 1,2-DCA, 1,2-DCP, and 1,1,2-TCA at concentrations comparable to those present in the DNAPL source zone area of the Brooklawn area of the PPI site.

*D. alkenigignens* IP3-3^T^ was isolated from groundwater contaminated with 1,2-DCA, 1,2-DCP, and 1,1,2-TCA at concentrations of 0.023 mM, 0.021 mM, and 0.010 mM, respectively ([Bowman et al., 2012]). Although initially isolated from an environment with much lower chlorinated solvent concentrations than *D. lykanthroporepellens* BL-DC-9^T^, results from the present study demonstrate that *D. alkenigignens* IP3-3^T^ can reductively dechlorinate 1,2-DCA, 1,2-DCP, and 1,1,2-TCA at concentrations comparable to *D. lykanthroporepellens* BL-DC-9^T^.

Although reports of pure cultures’ abilities to dehalogenate high concentrations of chlorinated alkanes are generally lacking in the literature, Marzorati et al. ([2007]) reported an enrichment culture referred to as 6VS (originating from groundwater in Italy where there was 1,2-DCA contamination for more than 30 years) that repeatedly dechlorinated 8 mM 1,2-DCA. Also, [Grostern and Edwards (2009]) described an enrichment culture, including *Dehalobacter* sp. and an *Acetobacterium* sp.*,* capable of dechlorinating 2 mM 1,2-DCA. Though not previously evaluated for chlorinated ethanes or propanes, previous research on chlorinated ethenes has shown that microbial populations reductively dechlorinating chlorinated aliphatic alkenes, particularly perchloroethene (PCE) and trichloroethene (TCE) can maintain their activity and increase contaminant dissolution rates ([Cope and Hughes, 2001]; [Dennis et al., 2003]; [Sleep et al., 2006]; [Yang and McCarty 2002]).

The toxicity of solvents to microorganisms has been previously correlated to hydrophobicity as measured by the log of octanol/water partition coefficients, log K_ow_ ([Sikkema et al., 1995]). Compounds with log K_ow_ in the range of 1.5 to 4 are generally toxic to microorganisms, with maximum toxicity exhibited by compounds with log K_ow_ between 2 and 4 ([Bowman et al., 2009]; [Inoue and Horikoshi, 1991]; [Kieboom and de Bont, 2000]; [Sikkema et al., 1994]; [Sikkema et al., 1995]). The adverse effects of 1,2-DCA, 1,2-DCP, and 1,1,2-TCA on reductive dechlorination by the bacterial strains tested here are consistent with these previous observations. For equal molar concentrations, 1,1,2-TCA [log K_ow_ 2.47, ([Alvarez and Illman, 2006])] was more inhibitory than 1,2-DCP [log K_ow_ 2.0, ([Alvarez and Illman, 2006])] which had a larger adverse effect than 1,2-DCA [log K_ow_ 1.48, ([Alvarez and Illman, 2006])].

The ability of *Dehalogenimonas* spp. to reductively dechlorinate high concentrations of halogenated alkanes has important implications for cleanup of contaminated soil and groundwater. Abiotic transformation of these chemicals in the environment is generally quite slow. For example, the environmental half-life of 1,2-DCA from abiotic transformation in water was estimated to be 50 years ([Vogel et al., 1987]). Unlike chlorinated ethenes (e.g., tetrachloroethene and trichloroethene), several of the polychlorinated ethanes and propanes, 1,2-DCA in particular, are resistant to transformation by zero-valent iron ([Sarathy et al., 2010]; [Song and Carraway, 2005]), limiting physicochemical remediation approaches for cleanup. The fact that *Dehalogenimonas* spp. are able to perform reductive dechlorination even in the presence of high concentrations of chlorinated compounds suggests that they may provide an important role in bioremediation.

## Abbreviations

1,2-DCA: 1,2-dichloroethane; 1,2-DCP: 1,2-dichloropropane; 1,1,2-TCA: 1,1,2-trichloroethane.

## Competing interests

The authors declare that they have no competing interests.

## Authors' contributions

JY conducted preliminary experiments to assess the range of chlorinated solvents dehalogenated by *D. lykanthroporepellens* BL-DC-9^T^. ADM and KSB carried out the final experiments with *D. alkenigignens* IP3-3^T^ and *D. lykanthroporepellens* BL-DC-9^T^. WMM and FAR guided the research. All authors contributed to data interpretation and writing of the manuscript. All authors read and approved the final manuscript.
